# Officers of the American Medical Association

**Published:** 1889-11

**Authors:** 


### Officers of the American Medical Association.

At the late meeting of the American Medical Association the following officers were elected, and appointments made:

President, E. M. Moore, of Rochester, N. Y.

Vice-Presidents, J.W. Jackson, of Missouri; W. W. Kimble, of Minnesota; J. H. Warren, of Massachusetts; T. B. Evans, of Maryland.

Permanent Secretary, Wm. B. Atkinson, of Philadelphia.

Treasurer, R. J. Dunglison, of Philadelphia.

Librarian, C. H. Kleinschmidt, of Washington, D. C.

Judicial Council, N. S. Davis, of Chicago; J. H. Brown, of Kentucky; Wm. Brodie, of Michigan; R. C. Moore, of Nebraska; —— Gillespie, of Tennessee; T. A. Forster, of Maine; J. B. S. Jones, of Georgia.

Trustees of the Journal, T. O. Hooper, of Arkansas; Alonzo Garcelon, of Maine; I. N. Love, of St. Louis; W. W. Dawson, of Cincinnati.

Address in State Medicine, A. L. Carroll, of New York.

Committee to fill vacancies in the appointments to deliver general addresses, Drs. William Brodie, J. H. Murphy, and I. G. Morris.

Place of next meeting, Nashville; time, the third Tuesday in May.

Chairman of the Committee of Arrangements, W. T. Briggs, of Nashville.

Assistant Secretary, G. C. Savage, of Nashville.

A board of physicians were inquiring into the state of mind of an alleged lunatic. "You told us just now," said the chairman, "that you were the Emperor Napoleon, and now you say you are the Duke of Wellington. Pray, explain yourself." "Quite right," returned the patient, cheerfully; "That was by a different mother." They did not ask him any more questions.

